# Supplementary material for: Transmembrane but not soluble helices fold inside the ribosome tunnel
Source: Nat Commun. 2018 Dec 7;9:5246. doi: 10.1038/s41467-018-07554-7 (PMC6286305; doi:10.1038/s41467-018-07554-7)
Supplement: Supplementary file 1 — Supplementary Information [file 41467_2018_7554_MOESM1_ESM.pdf]

Transmembrane but not soluble helices fold inside the ribosome tunnel

Bañó-Polo M., Baeza-Delgado C., Tamborero S., Hazel A., Grau B., Nilsson IM., Whitley P., Gumbart J.C., von Heijne G. and Mingarro I.

Supplementary Information

(contains Supplementary Figures 1-10 with Legends, Supplementary Table 1 and Supplementary References)

Supplementary Fig.1

VSV-G construct

…PGCSSGQACE **N**^{1}**ST**GVTYSNV EPSDFVQTFS RRNGGEATSG FFEVPMITSS IASFFFIIGL IIGLFLVLHM RLSERKE^{67}↲TLG DVT^{73}…

gp41 construct

…PGCSSGQACE **N**^{1}**ST**GVTYSNV EPSDFVQTFS RRNGGEATSG FFEVPMIKLF IMIVGGLVGL RIVFAVLSVV HMRLSER^{67}↲KET LGD^{73}…

NAGK construct

…PGCSSGQACE **N**^{1}**ST**GVTYSNV EPSDFVQTFS RRNGGEATSG FFEVPMISRD DAAQVAKVLS EALPYIRRFV HMRLSER^{67}↲KET LGD^{73}…

rib. L9 construct

…PGCSSGQACE **N**^{1}**ST**GVTYSNV EPSDFVQTFS RRNGGEATSG FFEVPMIKAL EAQKQKEQRQ AAEELANAKK HMRLSER^{67}↲KET LGD^{73}…

GpA construct

…PGCSSGQACE **N**^{1}**ST**GVTYSNV EPSDFVQTFS RRNGGEATSG FFEVPMITLI IFGVMAGVIG TILLISYGIK KKKHMRL^{67}↲SER KET^{73}…

M13 construct

…PGCSSGQACE **N**^{1}**ST**GVTYSNV EPSDFVQTFS RRNGGEATSG FFEVPMISYI GYAWAMVVVI VGATIGIKLF KHMRLSE^{67}↲RKE TLG^{73}…

p75 construct

…PGCSSGQACE **N**^{1}**ST**GVTYSNV EPSDFVQTFS RRNGGEATSG FFEVPMINLI PVYCSILAAV VVGLVAYIAF KRWNHMR^{67}↲LSE RKE^{73}…

Lep H1 construct

…PGCSSGQACE **N**^{1}**ST**GVTYSNV EPSDFVQTFS RRNGGEATSG FFEVPMIMAN MFALILVIAT LVTGILWCVH MRLSERK^{67}↲ETL GDV^{73}…

gp41 TM.5 construct

…PGCSSGQACE **N**^{1}**ST**GVTYSNV EPSDFVQTFS RRNGGEATSG FFEVPMIKLF IMIVGGLVGL RVRLSERKET LGDVTHR^{67}↲ILT VPI^{73}…

VSV-G TM.5 (TM10) construct

…PGCSSGQACE **N**^{1}**ST**GVTYSNV EPSDFVQTFS RRNGGEATSG FFEVPMITSS IASFFFIIHM RLSERKETLG DVTHRIL^{67}↲TVP IAQ^{73}…

VSV-G TM11 construct

…PGCSSGQACE **N**[1]**ST**GVTYSNV EPSDFVQTFS RRNGGEATSG FFEVPMITSS IASFFFIIGH MRLSERKETL GDVTHRI[67]↲LTV PIA[73]…

VSV-G TM14 construct

…PGCSSGQACE **N**[1]**ST**GVTYSNV EPSDFVQTFS RRNGGEATSG FFEVPMITSS IASFFFIIGL IIHMRLSERK ETLGDVT[67]↲HRI LTV[73]…

VSV-G TM17 construct

…PGCSSGQACE **N**[1]**ST**GVTYSNV EPSDFVQTFS RRNGGEATSG FFEVPMITSS IASFFFIIGL IIGLFHMRLS ERKETLG[67]↲DVT HRI[73]…

NAGK (-16)

…CE**Q**STGVTYS **N**[1]**ST**SDFVQTF SRRNGGEATS GFFEVPMISR DDAAQVAKVL SEALPYIRRF VHMRLSERKE TLGDVTH[67]↲RIL…

L9 (-16)

…CE**Q**STGVTYS **N**[1]**ST**SDFVQTF SRRNGGEATS GFFEVPMIKA LEAQKQKEQR QAAEELANAK KHMRLSERKE TLGDVTH[67]↲RIL…

Supplementary Figure 1. Truncated constructs analysed in this work. Amino acid sequences bridging the distance between the glycosylation acceptor site (**N**[1]ST) and the end of the translated region for distance 73 (d=73) are shown in single letter code. The last residue for truncates with 67 residues P-**N**ST (d=67) is indicated in each construct with an arrow (↲). Hydrophobic TM and soluble helical sequences are highlighted in yellow and grey boxes, respectively. Charged residues in these boxed regions are shown in red (Asp and Glu) or in blue (Lys and Arg).

Supplementary Fig.2

a

b

c

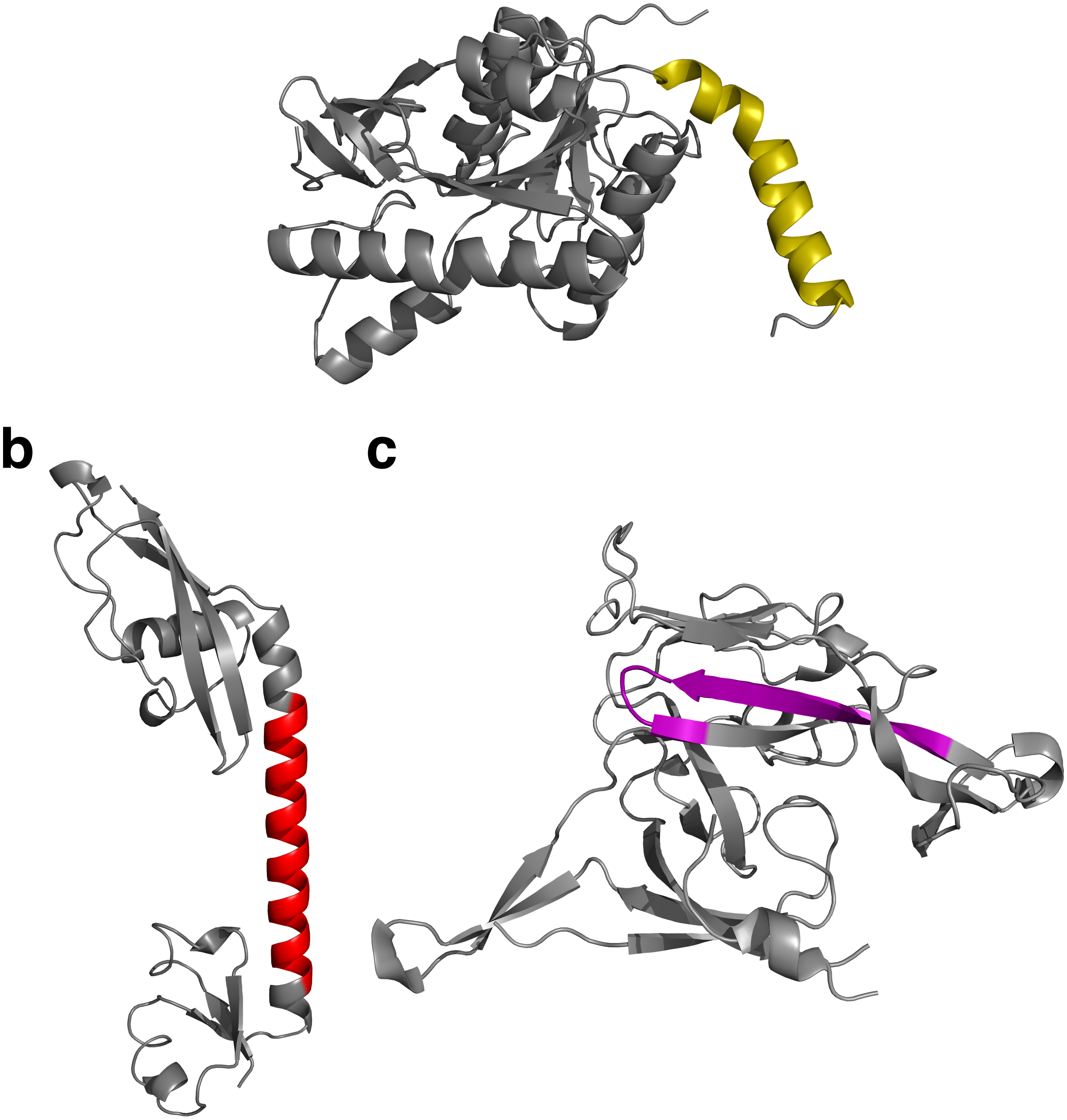

Supplementary Figure 2. **Cartoon representation of soluble proteins. a** N-AcetylGlutamate Kinase (NAGK) protein structure (PDB code 2BUF [https://www.rcsb.org/structure/2BUF]). The helical residues studied in this work are shown in yellow (from Arg5 to Val26). **b** Ribosomal L9 protein structure (PDB code 1DIV [https://www.rcsb.org/structure/1DIV]). The helical residues studied in this work are shown in red (from Lys45 to Lys67). **c** Signal Peptidase I P2 domain (Lep P2) structure (PDB code 1B12 [https://www.rcsb.org/structure/1B12]). The non-helical residues (63-73 in the ribosome-nascent chains) used in this work following the helical segments are shown in pink (from Arg223 to Thr235, Lep sequence). Pictures made with Pymol Molecular Graphics System (v1.7.0.0).

Supplementary Fig.3

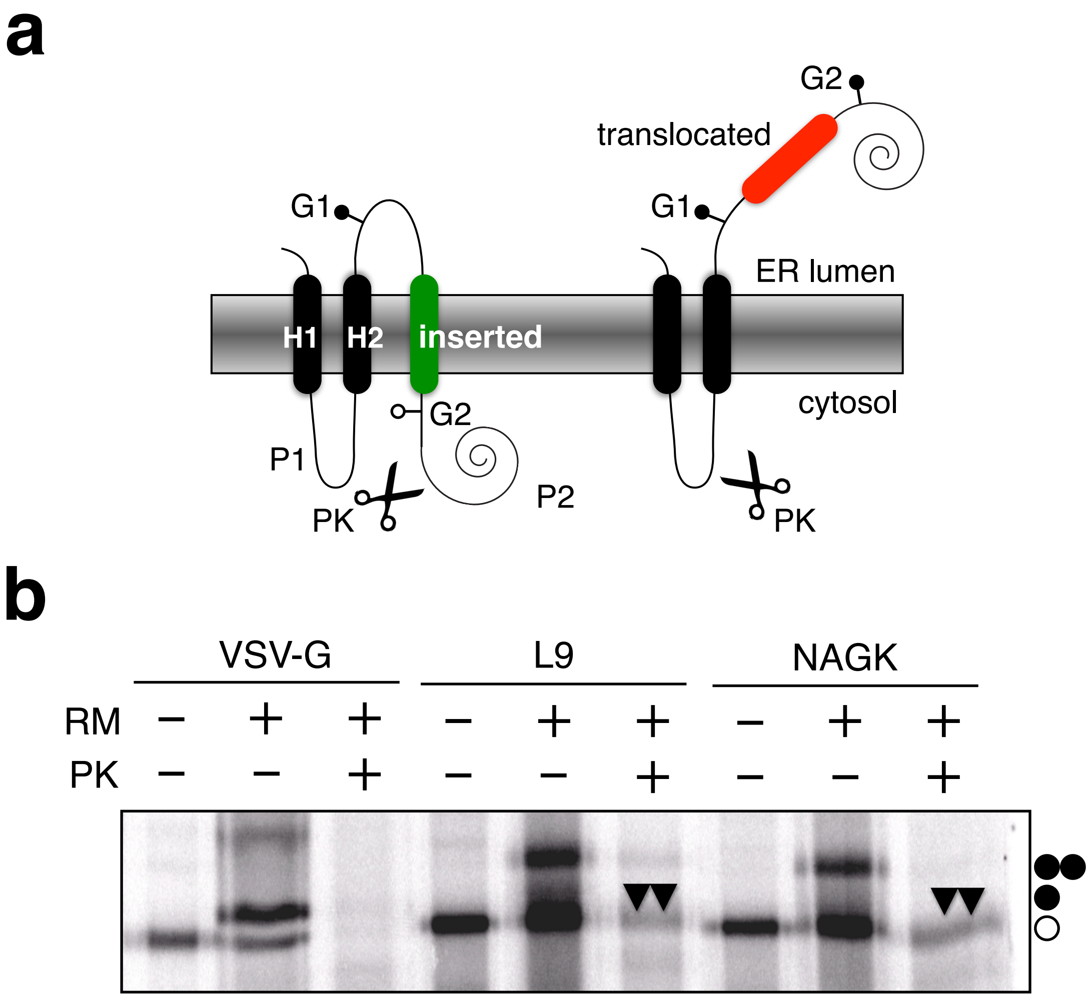

Supplementary Figure 3. Insertion of hydrophobic regions of VSV-G, L9 and NAGK into membranes using Lep as model protein. a Schematic representation of the Lep construct used to report insertion of hydrophobic region of VSV, and helical sequences of L9 and NAGK into endoplasmic reticulum membranes (top). The TM segment under investigation (coloured) was introduced into the P2 domain of Lep, flanked by two artificial glycosylation acceptor sites (G1 and G2). Recognition of the tested sequence as a TM domain by the translocon machinery results in the location of only G1 in the luminal side of the ER membrane, preventing G2 glycosylation (left). The Lep chimera will be doubly glycosylated when the sequence being tested is translocated into the lumen of the microsomes (right). **b** *In vitro* translation in the presence of membranes of the different Lep constructs. Constructs containing VSV-G TM (residues 463 to 482; lanes 1-3), L9 helix (residues 45 to 67; lanes 4-6) and NAGK helix (residues 5 to 26) were translated in the presence (+) and absence (−) of rough microsomes (RM) and proteinase K (PK). Bands of non-glycosylated proteins are indicated by a white dot; singly and doubly glycosylated proteins are indicated by one and two black dots, respectively. The protected doubly-glycosylated H2/L9/P2 and H2/NAGK/P2 fragments are indicated by two arrowheads.

Supplementary Fig.4

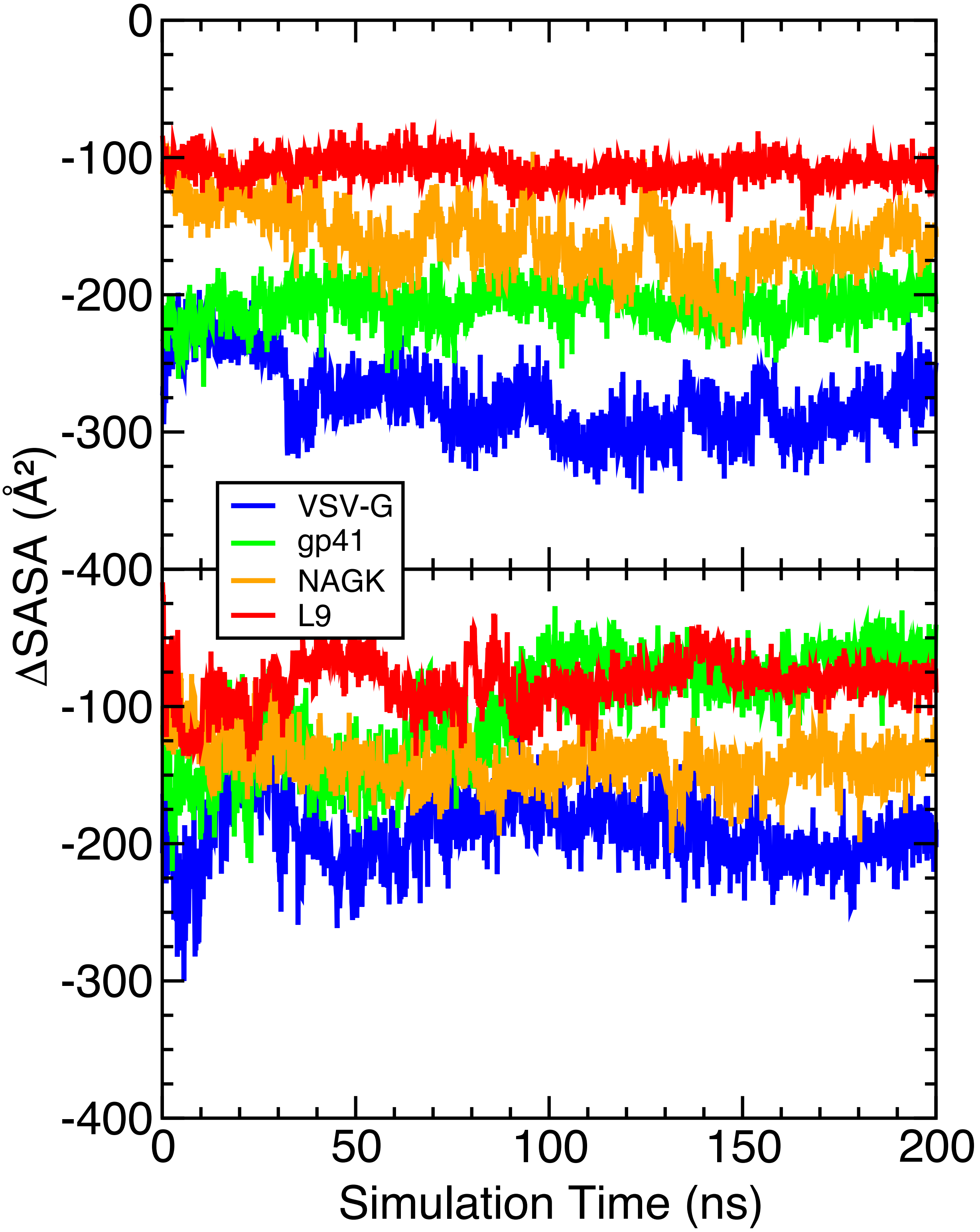

Supplementary Figure 4. Solvent accessible surface area (SASA) for TM and soluble sequences in the ribosome exit tunnel. ΔSASA (see Methods) for hydrophobic residues within nascent peptide sequences VSV-G (blue), gp41 (green), NAGK (orange), and L9 (red). More negative values indicate more hydrophobic contacts with the ribosome. (Top graph) α-Helical conformations. (Bottom graph) Extended conformations.

Supplementary Fig.5a

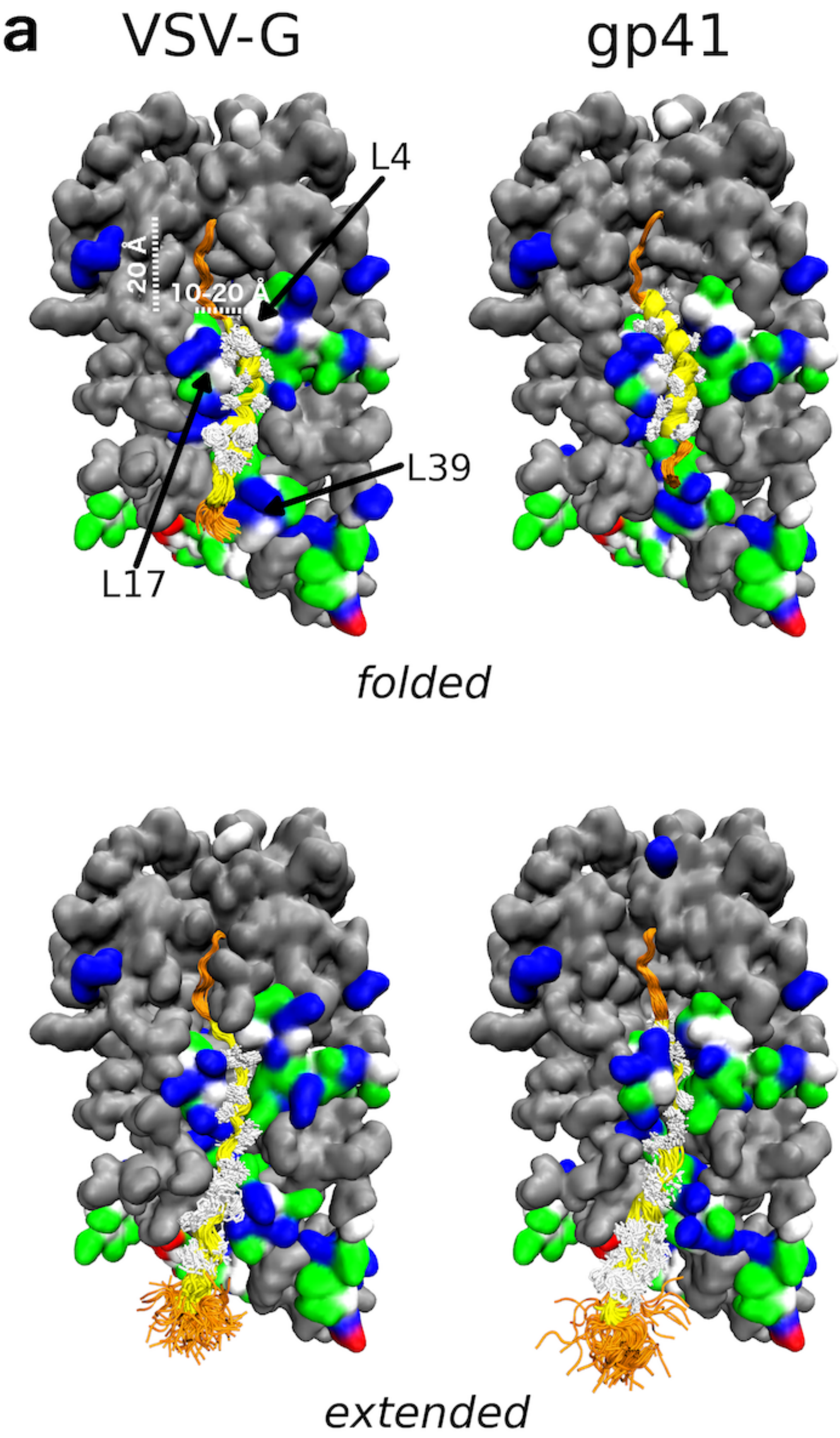

Supplementary Fig.5b

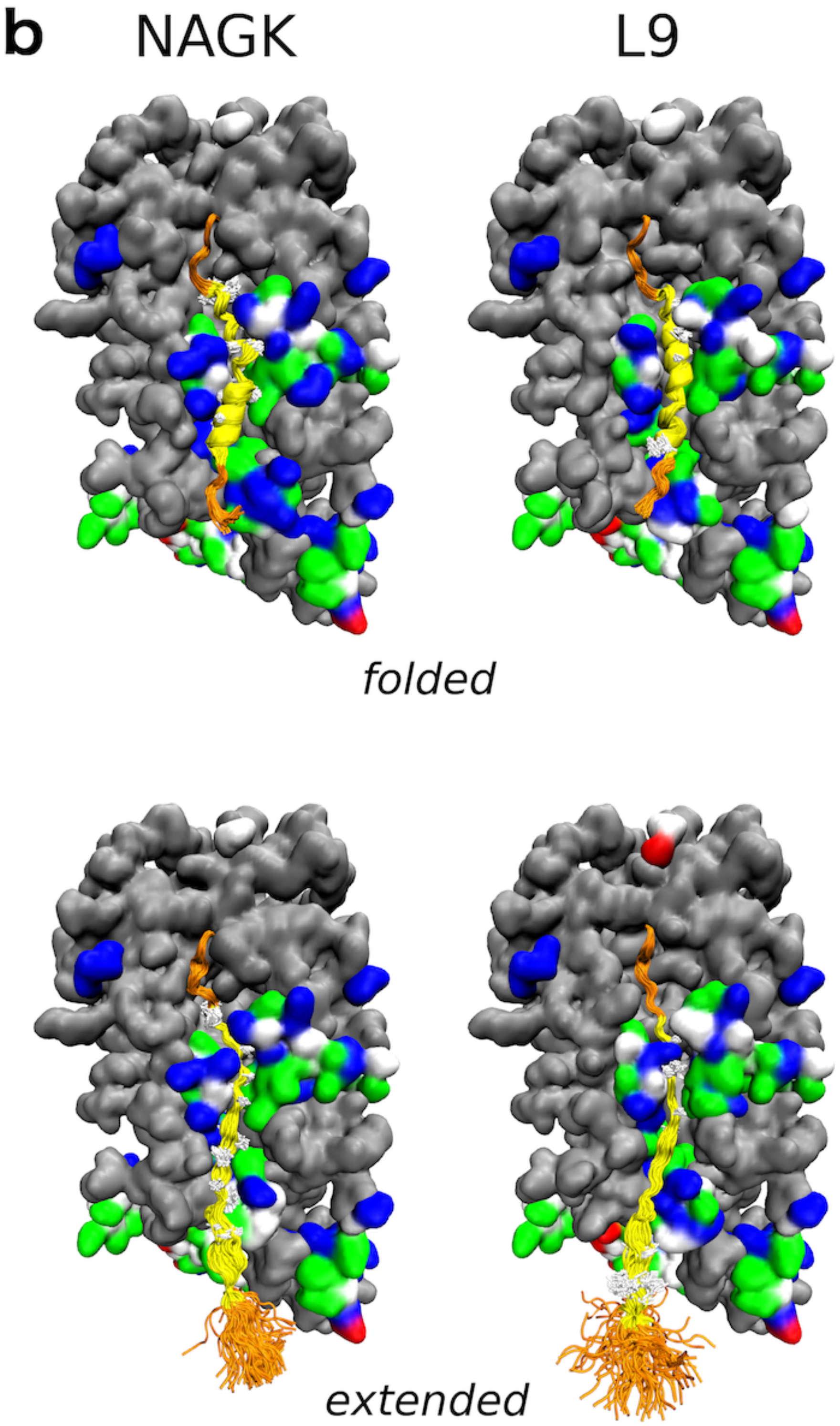

Supplementary Figure 5. **Ensemble of nascent peptide conformations within the ribosome exit tunnel. (a)** TM sequences VSV-G and gp41. **(b)** Soluble sequences NAGK and L9. The nascent peptide is shown in cartoon representation. Samples of the nascent peptide taken every 1 ns from the last 100 ns of each simulation are shown. Helical residues are colored yellow, and non-helical residues are colored orange. Hydrophobic side chains within the helical region are shown in licorice representation and colored white, with hydrogen atoms omitted. Ribosomal proteins are shown in a surface representation and colored by residue type: (white) hydrophobic, (green) polar, (blue) positively charged, and (red) negatively charged. Ribosomal proteins L4, L17, and L39 are labeled in (a). Ribosomal RNA is also shown in a surface representation and colored gray. Ribosomal atomic coordinates are taken from the final frame of each simulation.

Supplementary Fig.6

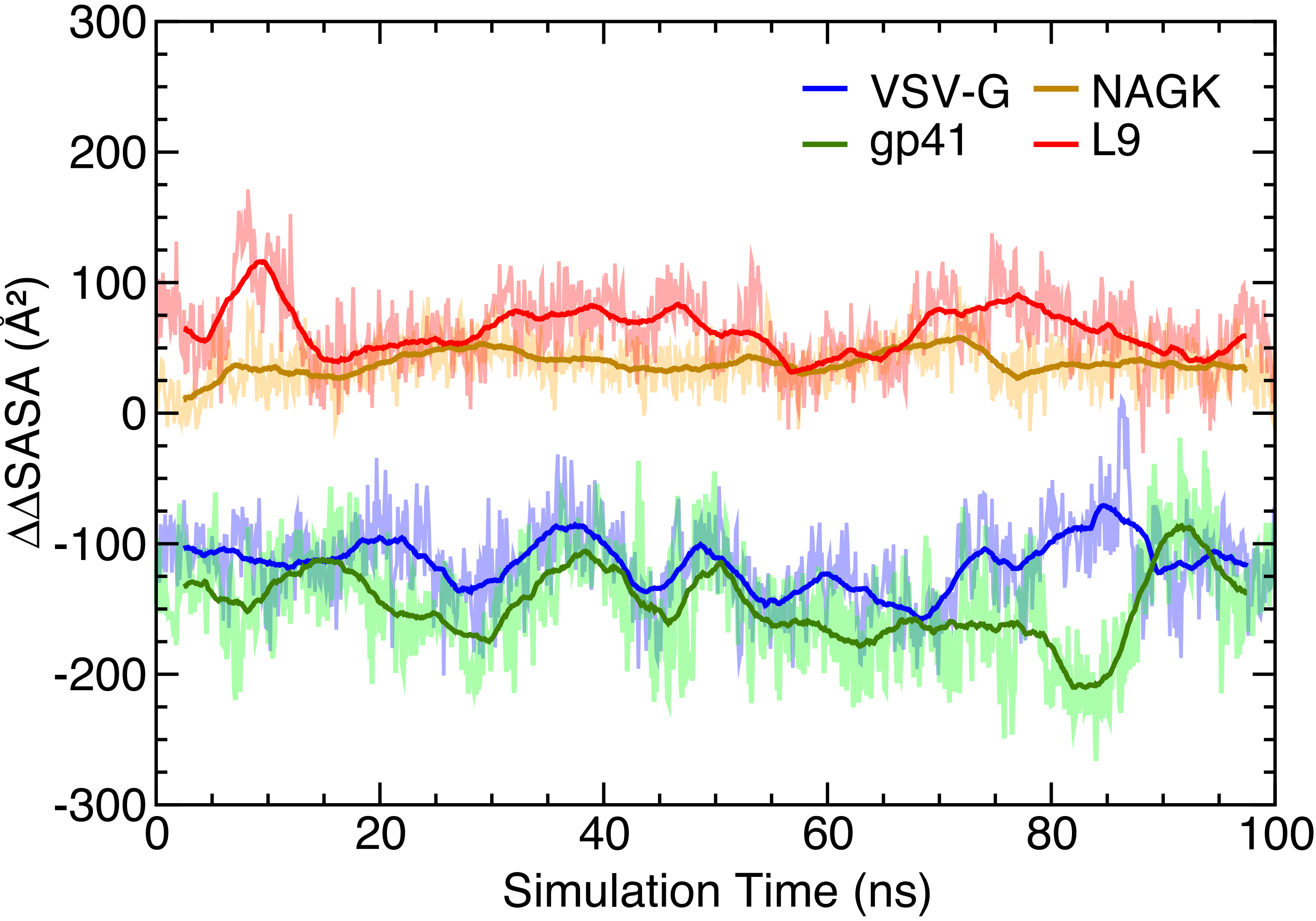

Supplementary Figure 6. ΔΔSASA of TM and soluble helical sequences within a bacterial (*E. coli*) ribosome. ΔΔSASA = ΔSASAfolded − ΔSASAextended (see Methods for the definition of ΔSASA). Bacterial ribosome and nascent polypeptide structures were modified from Gumbart *et al.* [1]. Simulations for helical and extended conformations were prepared in a similar manner to those of the human ribosome systems (see Methods).

Supplementary Fig.7

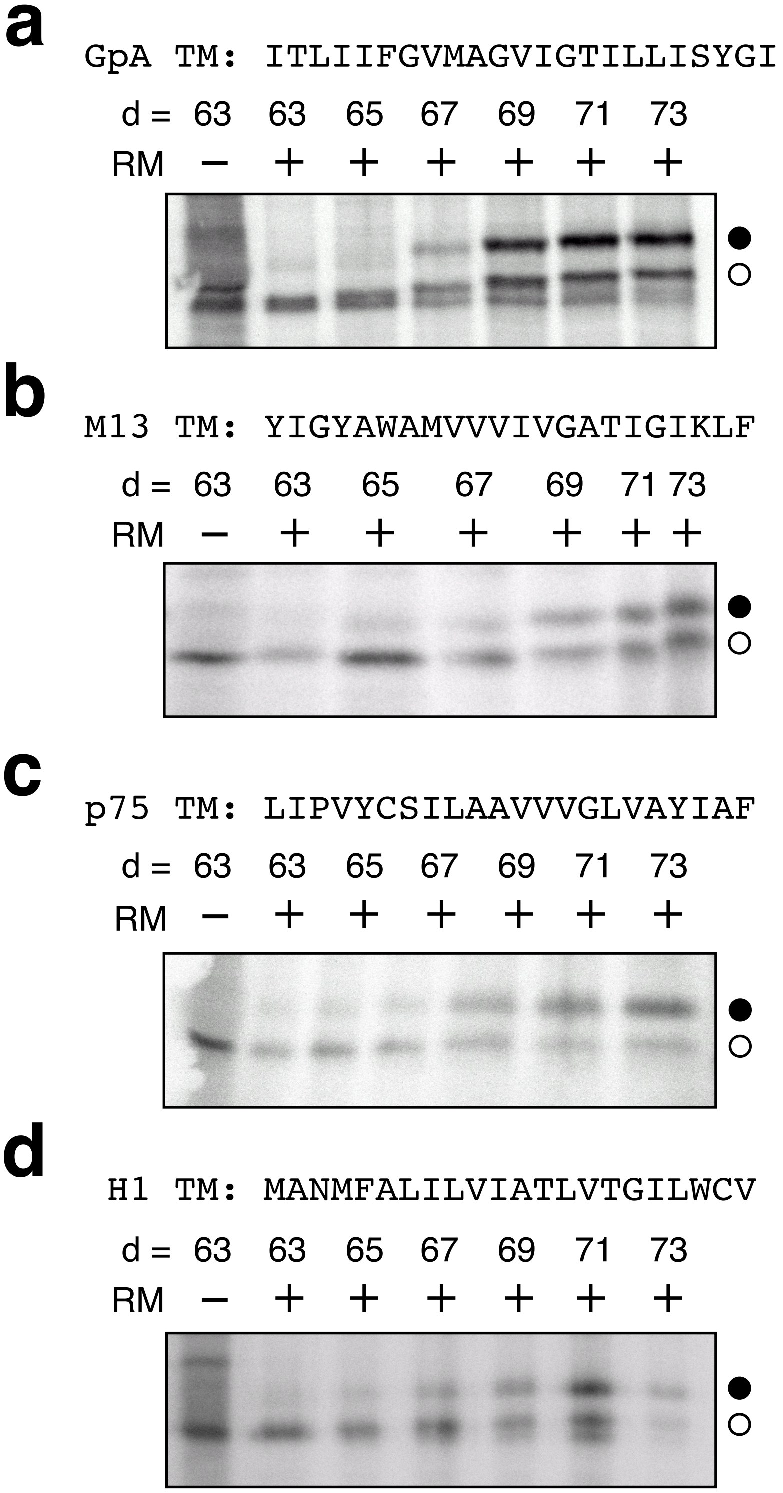

Supplementary Figure 7. **GpA, M13, p75 and H1 truncates containing TM helix sequences are folded inside the ribosomal exit tunnel.** The TM sequence used in each construct is shown on top of the respective SDS-PAGE autoradiography. Constructs harboring GpA (**a**), M13 coat protein (**b**), neurotrophin receptor p75 (**c**) and Lep H1 (**d**) TM sequences were translated *in vitro* in the absence (-) and presence (+) of rough dog pancreas microsomes (RM). The 3' codon in the truncated mRNA was placed *d* codons (distances 63 to 73) downstream of the Asn residue in the Asn-Ser-Thr glycosylation acceptor site. Non-glycosylated and glycosylated molecules are indicated by white and black dots, respectively.

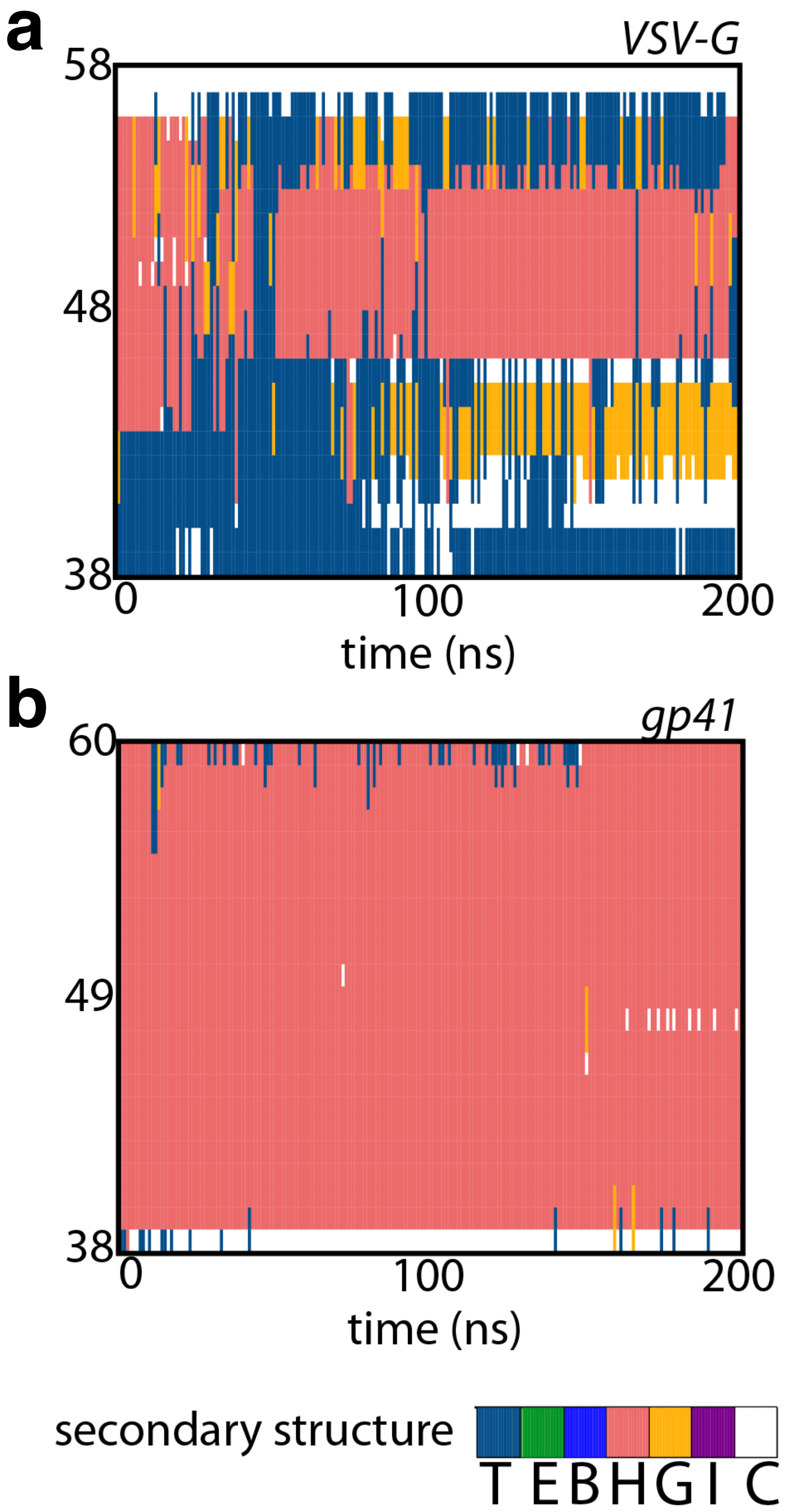

Supplementary Figure 8. Secondary structure of nascent peptides within the ribosomal exit tunnel. Secondary structure of TM residues 38 to 58 for VSV-G (**a**) and 38 to 60 for gp41 (**b**) of the nascent peptides in the helical conformation within the ribosomal exit tunnel during 200ns of equilibrium molecular dynamics simulations. Secondary structure codes: (T) turn, (E) extended, (B) isolated bridge, (H) α-helix, (G) 310-helix, (I) π-helix, and (C) coil (random or unstructured).

Supplementary Fig.9

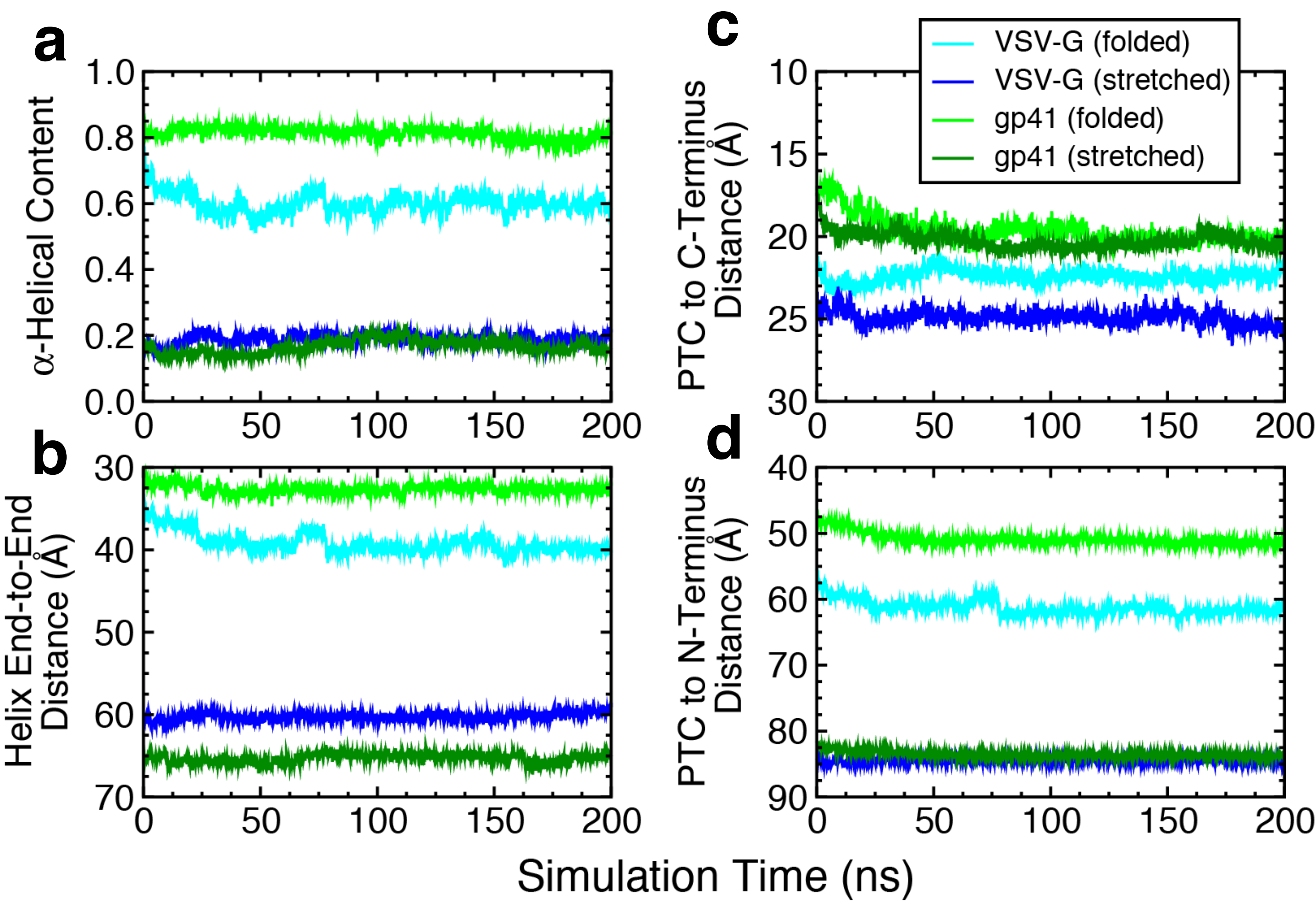

Supplementary Figure 9. Compactness of the TM segments VSV-G and gp41 in simulations in the mammalian ribosome PET. In all panels, lighter colors are used for the folded state and darker colors for the fully extended state. **a** α-helical content as measured in NAMD. Briefly, this collective variable takes as input both the angle between every three successive Cα atoms and the strength of hydrogen bonds between backbone N and O atoms of every i and i+4 residues; 1.0 is a perfect α-helix and 0.0 is no helical content whatsoever. **b** End-to-end distance over time of the helical residues, i.e., residues 38-58 for VSV-G and 38 to 60 for gp41. **c-d** Distance from the P-site (PTC) to the C-terminus **(c)** and to the N-terminus **(d)**.

Supplementary Fig.10

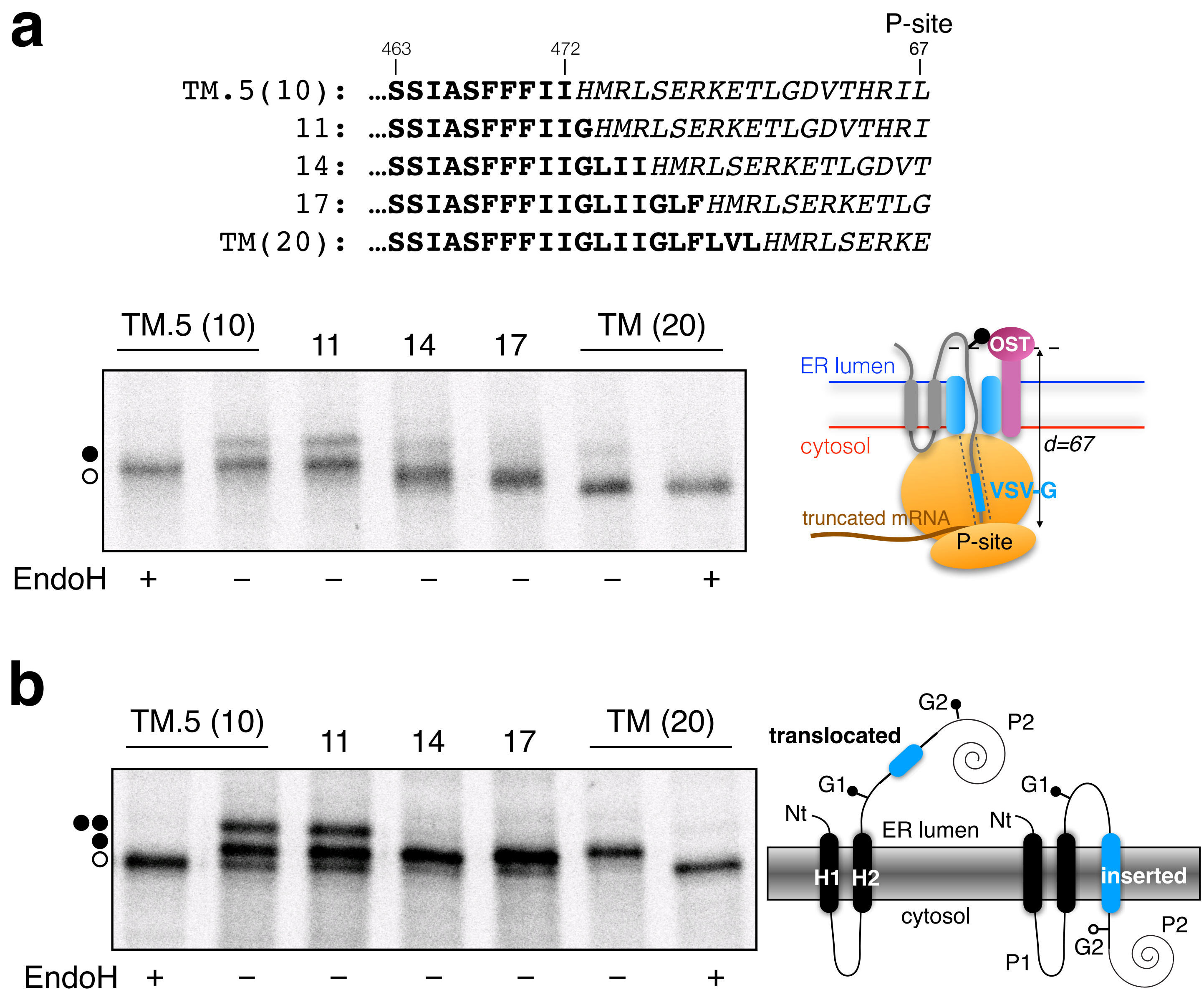

Supplementary Figure 10. At least 14 VSV-G hydrophobic residues are need it to compact within the ribosome exit tunnel and to insert into microsomal membranes. a *In vitro* translation in the presence of membranes of the different constructs. Truncated mRNAs containing VSV-G TM (residues 463 to 472, TM.5(10); 463-473, 11; 463-476, 14; 463-479, 17; and 463-482, TM(20)) were translated in the presence of rough microsomes and in the presence (+) or in the absence (-) of Endoglycosydase H (EndoH), a glycan-removing enzyme. The 3' codon in the truncated mRNAs were placed 67 residues downstream of the Asn residue in the Asn-Ser-Thr glycosylation acceptor site. Bands of non-glycosylated and glycosylated proteins are indicated by a white and a black dot, respectively. Cartoon shown on the right. Top: the hydrophobic residues from VSV-G TM segment and the flanking Lep-derived sequences used in each construct are shown in bold and italic, respectively. **b** I*n vitro* protein translation in the presence of microsomes and in the presence (+) or absence (-) of EndoH of VSV-G TM containing Lep-derived constructs. Non-glycosylated protein bands are indicated by a white dot; single and double glycosylated protein bands are indicated by one or two black dots, respectively. Cartoon shown on the right.

Supplementary table 1. Primers used in this study

	Clonations	
**Gp41**	gp41 Forward	gctatgatcaaattattcataatgata
	gp41 Reverse	cgatcatatgcactacagaaagtacagc
**L9**	L9 Forward	gctatgatcaaagcgctcgaggcgcaa
	L9 Reverse	cgatcatatgtttttttcgcattcgccag
**NAGK**	NAGK Forward	gctatgatcagtcgcgatgacgccgcccag
	NAGK Reverse	cgatcatatggacgaagcggcggatgta
**VSV**	VSV Forward	gctatgatcacaagctctattgcaagcttt
	VSV Reverse	cgatcatatggagaaccaagaatagtcc
**Gp41**	GpA Forward	gctatgatcacactcattatttttggggtg
	GpA Reverse	cgatcatatgcttcttctttttaatacc
**L9**	M13 Forward	gctatgatcagctatatcggttatgcgtgg
	M13 Reverse	cgatcatatgcttaaacagcttgatacc
**NAGK**	p75 Forward	gctatgatcaacctcattcctgtctat
	p75 Reverse	cgatcatatggttccacctcttgaaagc
**VSV**	H1 Forward	gctatgatcatggcgaatatgtttgcc
	H1 Reverse	cgatcatatgcacgcaccataaaatgcc

	Site directed mutagenesis	
**VSV**	VSV-LL Forward	gcaagctttttctttctgctgggcctgatcattgga
	VSV-LL Reverse	tccaatgatcaggcccagcagaaagaaaaagcttgc
	VSV-AA Forward	gcaagctttttctttgcagcgggcctgatcattgga
	VSV-AA Reverse	tccaatgatcaggcccgctgcaaagaaaaagcttgc
	VSV-GG Forward	gcaagctttttctttggcgggggcctgatcattgga
	VSV-GG Reverse	tccaatgatcaggcccccgccaaagaaaaagcttgc
	VSV-PP Forward	gcaagctttttctttcccccaggcctgatcattgga
	VSV-PP Reverse	tccaatgatcaggcctgggggaaagaaaaagcttgc
	VSV-KK Forward	gcaagctttttctttaaaaagggcctgatcattgga
	VSV-KK Reverse	tccaatgatcaggcccttttaaagaaaaagcttgc
	VSV-DD Forward	gcaagctttttctttgacgacggcctgatcattgga
	VSV-DD Reverse	tccaatgatcaggccgtcgtcaaagaaaaagcttgc

	Site directed mutagenesis deletion	
**VSV**	VSV TM.5 Forward	ctattgcaagcttcttcttcatcatacatatgcgtctttccgagcg
	VSV TM.5 Reverse	cgctcggaaagacgcatatgtatgatgaagaagaagcttgcaatag
	VSV TM.5 (11) Forward	tgcttcttcttcatcataggccatatgcgtctttccgag
	VSV TM.5 (11) Reverse	ctcggcaagacgcatatggcctatgatgaagaagaagct
	VSV TM.5 (14) Forward	agcttcttcttcatcataggcctgatcattcatatgcgtctttccgag
	VSV TM.5 (14) Reverse	ctcgaaaagacgcatatgaatgatcaggcctatgatgaagaagaagct
	VSV TM.5 (17) Forward	ctgctcattggactattccatatgcgtctttccgag
	VSV TM.5 (17) Reverse	ctcggaaagacgcatatggaatagtccaatgatcag
**gp41**	gp41 TM.5 Forward	ggaggcttggtaggtttaagagtacgtctttccgagcgtaaagag
	gp41 TM.5 Reverse	ctctttacgctcggaaagacgtactcttaaacctaccaagcctcc

	Truncates	
**All**	Forward T7	atagtataatacgactcactataggaaaccaccatggcgaatatg
**gp41**	Reverse Gp41 d63	acgcatatgcactac
	Reverse 1 d65	ggaaagacgcatatg
	Reverse 1 d67	acgctcggaaagacg
	Reverse 1 d69	ctctttacgctcgga
	Reverse 1 d71	cagtgtctctttacg
	Reverse 1 d73	atcacccagtgtctctttacgctcggaaagacgca
**L9**	Reverse L9 d63	acgcatatgtttttt
	Reverse 1 d65	ggaaagacgcatatg
	Reverse 1 d67	acgctcggaaagacg
	Reverse 1 d69	ctctttacgctcgga
	Reverse 1 d71	cagtgtctctttacg
	Reverse 1 d73	atcacccagtgtctctttacgctcggaaagacgca
**NAGK**	Reverse NAGK d63	acgcatatggacgaa
	Reverse 1 d65	ggaaagacgcatatg
	Reverse 1 d67	acgctcggaaagacg
	Reverse 1 d69	ctctttacgctcgga
	Reverse 1 d71	cagtgtctctttacg
	Reverse 1 d73	atcacccagtgtctctttacgctcggaaagacgca
**VSV**	Reverse 2 d63	ggaaagacgcatatg
	Reverse 2 d65	acgctcggaaagacg
	Reverse 2 d67	ctctttacgctcgga
	Reverse 2 d69	cagtgtctctttacg
	Reverse 2 d71	atcacccagtgtctctttacgctcggaaagacgca
	Reverse 2 d73	cgtcacatcacccagtgtctctttacg
**GpA**	Reverse GpA d63	cttcttctttttaat
	Reverse GpA d65	catatgcttcttctt
	Reverse GpA d67	aagacgcatatgctt
	Reverse GpA d69	ctcggaaagacgcat
	Reverse GpA d71	tttacgctcggaaag
	Reverse GpA d73	tgtctctttacgctc
**M13**	Reverse M13 d63	catatgcttaaacag
	Reverse M13 d65	aagacgcatatgctt
	Reverse M13 d67	ctcggaaagacgcat
	Reverse M13 d69	tttacgctcggaaag
	Reverse M13 d71	tgtctctttacgctc
	Reverse M13 d73	acccagtgtctcttt
**p75**	Reverse p75 d63	ccacctcttgaaagcaata
	Reverse p75 d65	atggttccacctctt
	Reverse p75 d67	acgcatatggttcca
	Reverse p75 d69	ggaaagacgcatatg
	Reverse p75 d71	acgctcggaaagacg
	Reverse p75 d73	ctctttacgctcgga
**H1**	Reverse H1 d63	aagacgcatatgcac
	Reverse H1 d65	ctcggaaagacgcat
	Reverse H1 d67	tttacgctcggaaag
	Reverse H1 d69	tgtctctttacgctc
	Reverse H1 d71	acccagtgtctcttt
	Reverse H1 d73	cacatcacccagtgt
**gp41 TM.5**	Reverse gp41 TM.5 d67	gcggtgcgtcacatc
**VSV TM.5**	Reverse VSV 10 d67	cagaatgcggtgcgt
	Reverse VSV 11 d67	aatgggtgcgtcacatc
	Reverse VSV 14 d67	cgtcacatcacccagtgtctctttacg
	Reverse VSV 17 d67	acccagtgtctctttacg

Supplementary References

1. Gumbart, J., Schreiner, E., Wilson, D. N., Beckmann, R. & Schulten, K. Mechanisms of SecM-mediated stalling in the ribosome. *Biophys J* **103**, 331-341 (2012).
